# A GP caregiver needs toolkit versus usual care in the management of the needs of caregivers of patients with advanced cancer: a randomized controlled trial

**DOI:** 10.1186/1745-6215-11-115

**Published:** 2010-11-29

**Authors:** Geoffrey Mitchell, Afaf Girgis, Moyez Jiwa, David Sibbritt, Letitia Burridge

**Affiliations:** 1 Discipline of General Practice, University of Queensland Medical School, Herston Road, Herston, QLD, Australia; 2 Centre for Health Research & Psycho-oncology (CHeRP), Cancer Council NSW, University of Newcastle & Hunter Medical Research Institute, Level 2, David Maddison Building, Callaghan, NSW, Australia; 3 Curtin Health Innovation Research Institute, GPO Box U1987, Perth, WA, Australia; 4 School of Medicine & Public Health, University of Newcastle, Level 3, David Maddison Building, Callaghan, NSW, Australia

## Abstract

**Background:**

Caring for a person with progressive cancer creates challenges for caregivers. However the needs of caregivers are often not assessed or recognised by health care providers. Research is also lacking in this area, with little knowledge relating to effective strategies to address the specific needs of caregivers. This paper outlines a study protocol aimed at developing and evaluating the effectiveness of a general practice-based intervention to better meet the needs of caregivers of patients with advanced cancer.

**Methods/Design:**

Two hundred and sixty caregivers will be randomised into each of two arms of the intervention (520 participants in total) through patients with advanced cancer attending medical and radiation oncology outpatient clinics at two tertiary hospital sites. Consenting caregivers will be followed up for six months, and telephone surveyed at baseline, 1, 3 and 6 months following their entry into the study or until the patient's death, whichever occurs first. Assessment and management of the unmet needs of caregivers in the intervention arm will be facilitated through a specifically developed general practice-based strategy; caregivers in the control group will receive usual care. Qualitative interviews will be conducted with a sample of up to 20 caregivers and 10 GPs at the conclusion of their participation, to explore their views regarding the usefulness of the intervention.

**Discussion:**

This study will determine whether systematic assessment of caregiver needs supported by caregiver-specific information for General Practitioners is effective in alleviating the unmet needs experienced by caregivers caring for patients with advanced cancer.

**Trial registration number:**

ISRCTN: ISRCTN43614355

## Background

As our ageing population increases, so too does our reliance on the support and care provided by informal caregivers. In 2001, 12.5% of the Australian population was aged over 65, and this is estimated to rise to 25% by 2052 [1]. The number of people with a chronic or life-threatening disease will increase and with it, the absolute numbers of patients requiring palliative care (PC). Although 80% of patients express a wish to die at home, only a third actually do so [2]. However, 90% of patient care in the final year of life occurs at home [3], with many patients moving to an inpatient setting in the final days of life. Hence, much of the burden of care giving is managed in the community, with the greatest burden falling on primary caregivers [4]. Cancer is one of the 10 most common health conditions in receipt of informal care giving in Australia. Care may be equivalent to a full-time job, with 20% of caregivers providing full-time or constant care [5].

Caring for a patient with PC needs carries a number of implications. It is often provided by people who are themselves elderly and/or ill, and care giving may exacerbate the illness burden. Caregivers of patients receiving PC have lower quality of life (QoL-impairment in physical functioning, general health, and vitality) and worse overall physical health than caregivers of patients receiving curative or active treatment [6]. As patients deteriorate physically, caregiver QoL worsens, suggesting a greater need for support at this time [7]. Furthermore, whilst many caregivers feel positively about care giving and derive deep satisfaction in this role, caregivers may also experience a number of physical and psychosocial issues, including reduced social contact, significant financial burden, sadness, anger or resentment which may increase the risk of psychiatric morbidity and complicated grief [4]. Additionally, caregivers report a number of unmet needs across a variety of areas, including: information, communication, and support from services [8]. Family members whose own needs are not identified and addressed early have greater needs, less trust of and confidence in the health care system, and cope more poorly in the later stages, than families who have been informed and supported throughout the course of the illness [9].

However, despite the shift to include family members and primary caregivers as active components in PC [10], it is still common that the primary focus of the caregiver and of health professionals is on the needs and comfort of the patient, meaning that caregiver needs and distress may be considered secondary to the patient's and may be overlooked [11]. For example, in 2003/4, the patient was the client in 98.3% of referrals to Home and Community Care services, yet caregivers are a target group of that program [12]. It has also been suggested that caregivers may be reluctant to raise their own needs with their health care provider, as they do not wish to put their own issues before the patient's or bother the health care professional [13]. On the other hand, health care professionals are often working under tight time restraints, with the average Australian general practice consultation lasting only 14.6 minutes [14], making it difficult to assess both the patient and caregivers needs in one appointment.

Although international literature highlights GPs' variable levels of knowledge about PC and symptom control [15] and their lower perceived levels of competence in these areas compared to specialist PC services, GPs are in an optimal position to evaluate and assess the needs of caregivers. GPs are usually the first point of contact for patients and their caregivers and generally have an established relationship with the patients with palliative care needs as well as having an important contextual knowledge of the family and of the illness. Canadian research indicates that palliative care patients with a regular GP are less likely to seek care from emergency departments [16], and are less likely to die in hospital [17].

Regardless of our knowledge as to the importance of evaluating and addressing caregiver needs and the optimal position that GPs hold for doing this, there is a lack of research investigating the most effective strategies to meet the needs of caregivers. A systematic review of interventions designed to alleviate caregiver burden found only nine such studies [18]. Interventions reported included home care, home respite services, nurse practical and emotional support, and group support, with no reported interventions based in general practice.

Considering the past evidence and lack of research aimed at addressing the unmet needs of caregivers, we will be implementing an intervention which focuses specifically on the needs of caregivers, using GPs as the point of intervention. Specifically, we propose conducting a randomized control trial (RCT), which utilises a GP Caregiver Needs Toolkit (hereafter referred to as the GP Toolkit). The GP Toolkit will contain two resources, which will assist GPs in systematically identifying and addressing the unmet needs of caregivers. The proposed study will build on existing work aimed to improve needs-based PC, and will include a) developing and evaluating the effectiveness of a general practice-based intervention to better meet the needs of caregivers caring for patients with advanced cancer; and b) assessing the efficacy of the intervention in reducing caregivers' reported number and levels of unmet needs when compared to usual care.

### Study aims and hypotheses

The overall aims of the proposed study are to:

1. Use randomized controlled methods to assess the efficacy of the systematic utilisation of a GP Toolkit in reducing caregivers' reported number and level of unmet needs.

2. Evaluate the acceptability of the intervention for GPs and caregivers.

For the proposed study it is hypothesised that the number and levels of unmet needs of caregivers of patients with advanced cancer will be significantly lower in caregivers whose needs are systematically assessed using a needs assessment tool and then addressed by their GP, compared with caregivers receiving usual care.

## Methods/Design

### Study design

The proposed study will use a mixed methods design comprising a RCT and a qualitative evaluation. Participating caregivers will be surveyed using a Computer Assisted Telephone Interview (CATI) at baseline, 1, 3 and 6 months from entry into the study, or until the patient's death, if this occurs first. Assessment and management of the unmet needs of caregivers in the intervention arm will be facilitated through the utilisation of the GP Toolkit resources by both the caregiver and GP following their entry into the study and at 3 months, while the caregivers in the control group will receive usual care. Ethics approval has been obtained from The University of Queensland and Princess Alexandra Hospital (Queensland), and the University of Newcastle (New South Wales). All participants will provide voluntary written consent.

### Setting

The foundation of the Australian health system rests on primary care delivered by GPs who accept unreferred patients and are the first point of contact for most health care problems including prevention and health promotion activities. Over time, the GP builds up a comprehensive knowledge of their patients' medical and psychosocial circumstances, and that of their family. Approximately 80% of all Australians visit a GP at least once per annum. In 2002-03, the Australian population of approximately 20 million people generated 89,832,327 unreferred attendances, ie approximately 4.5 per person per annum [19]. Should specialist medical or allied health services be required, GPs are usually responsible for referring the patient appropriately. This arrangement is funded by a government administered universal health insurance system, which supports a fee-for-service remuneration structure. In 1999, the Australian Government instituted a second form of funding, which encouraged more comprehensive care planning for patients with complex health problems or chronic diseases. This Program, the Enhanced Primary Care (EPC) Program [20], encompasses a range of initiatives that encourage multidisciplinary assessment and management of these problems, and are the basis of the intervention below.

### Participants

### Sample and recruitment

A consecutive sample of caregiver participants will be recruited through Medical and Radiation Oncology services at two major tertiary hospital sites in Brisbane, Australia, in liaison with clinic personnel. These sites provide oncology services to approximately 2.5 million people in southern Queensland, Australia. There are approximately 2,200 GPs in this region. To be eligible to participate in the proposed study, caregivers must: (1) be a nominated caregiver of a patient with a diagnosis of advanced cancer (i.e. cancer that is no longer amenable to cure, with either extensive local or regional spread or metastatic disease); (2) be aged 18 years or older; (3) live within the greater Brisbane area (population approximately 2.0 million); and (4) be able to understand English sufficiently to complete questionnaires and telephone interviews. For practical reasons, the recruitment procedures will be adapted to suit the two recruitment sites. At one site, potentially eligible caregivers of patients attending oncology services will be identified by trained and experienced project personnel who will explain the study to them. Interested caregivers will be offered a flier with a brief overview of the study in plain language, and will be invited to ask the patient's doctor to confirm their eligibility on the flier. The eligibility criteria will be discreetly displayed for reference in each consulting room. Interested and eligible caregivers will be given an information pack to read and will be asked for their contact details for follow-up a week later by a project staff member, to answer any questions and ascertain their response. The information pack will inform caregivers about the study and invite them to return a signed consent form. At the second site, the Clinical Trials Co-ordinator will identify eligible caregivers in liaison with other clinic personnel, and will approach these caregivers to explain the study in plain language. Those who are eligible and interested will be offered the information pack to read, and will be followed up a week later by the Co-ordinator or a project staff member to answer any questions and ascertain their response.

GPs nominated by participating caregivers in the intervention group will be approached by research staff, who will inform them that their patient has agreed to participate, and seeking their willingness to participate.

### Randomization

Upon receiving the signed consent form, one project staff member will randomly allocate participating caregivers into the intervention or control group if their usual GP does not already have a caregiver patient in the study. Randomization will be conducted off-site using computer-generated random number tables and permuted block randomisation with blocks of four participants. However, if the caregiver's usual GP already has patients in the study, s/he will be allocated to that GP's study group, to minimise contamination between the two study arms. The risk of contamination is because the GPs of caregivers randomized to the intervention group have access to several interventions aimed at supporting the caregiver. (See *Intervention *below) There is therefore a high probability that a subsequent caregiver of that GP, randomized to the usual care group, will be offered different care to a caregiver of a GP who has not received training. Based on a previous study of similar populations, we expect that most GPs will have only one participant, and clustering will be minimal [21,22]

### Intervention

The GP Toolkit comprises two resources. The first resource will be a Needs Assessment Tool - Caregivers (NAT-C), which is a two-page needs assessment tool provided to intervention group caregiver participants to self-assess their unmet needs across a number of domains, including physical and psychological well-being, as well as spiritual, existential, social, financial and legal concerns. The tool can be completed by the caregiver or by the GP and caregiver together. The development of the NAT-C was based on a comprehensive literature review and was modelled on a pre-existing needs assessment tool, the Needs Assessment Tool: Progressive Disease - Cancer (NAT: PD-C), which has been extensively pilot-tested and validated [23,24]. Note that the NAT-C is not a validated measure of needs, rather a checklist that enables the content of the consultation to be focussed quickly on relevant matters.

The second resource is a folder of complementary materials for GPs, outlining the types of problems that might arise for caregivers and linking GPs to evidence--based information and other suggested strategies and resources that might help address problems identified at the consultation. These strategies include utilising the relevant EPC options, and accompanying documentation to facilitate their use. The resource materials will include brief descriptions and contact details of commonly used information services, such as Palliative Care Australia and State-based Cancer Council hotline resources for patients and caregivers. This resource will be supplied to GPs in a folder of indexed sections as well as in electronic format on a CD.

### Procedure

All participating caregivers will be mailed a copy of the CATI survey questions on entry to the study, for reference during the CATI phone calls. These interviews will be conducted by trained and experienced personnel, who will be blinded to the group assignment of participants. CATI data will be collected from control and intervention group caregivers at the same four time points. Baseline CATIs occur before the intervention group caregivers consult the GP. Apart from these interviews, the control group will not be subject to any further intervention from the project.

Intervention group caregivers will also receive a triplicate copy of the NAT-C, with a covering letter to explain the purpose of the NAT-C, to ask caregivers to complete this form and to attend their GP with the completed form as soon as practicable to discuss their needs, with a target of between one and two weeks to allow the GP to first receive an educational visit. At three months following entry into the study, intervention group caregivers will be asked to complete a second NAT-C and discuss it with their GP, to identify and deal with changing needs as the patient's disease progresses.

GPs of the intervention caregivers will be individually visited once by research staff as soon as possible after participant recruitment and allocation to the intervention group, to explain the use of all elements of the GP Toolkit. The research team will follow up GPs by phone to provide any further support required and to ensure the intervention is fully implemented. The frequency of these calls will be tailored to the needs of individual GPs. GPs with more than one caregiver participant will receive a visit only after the first participant is recruited. GPs will be encouraged to use the NAT-C as a guide to discussing the needs nominated by the caregiver, and to planning a course of action. There are no prescribed interventions for given identified needs. Any agreed actions are entirely up to the treating doctor and the caregiver.

Intervention patients who choose not to complete a NAT-C will still be encouraged to visit their GP, who may also choose to use the copy of the NAT-C in the GP Toolkit to guide the discussion, or not. The GP and the caregiver may choose to complete the NAT-C together.

### Outcome measures

A CATI survey will be conducted with caregivers at baseline (t0), one month (t1), three months (t2) and six months (t3) after their recruitment into the study. Demographic characteristics and aspects of the caregiving role will be assessed using questions previously utilised in a trial of health professional investigating the uptake of the *Palliative Care Needs Assessment Guidelines *[25] and NAT: PD-C [23,24].

The outcome variables to be assessed will include: unmet needs, anxiety, depression, QoL, coping style and financial impact. All of these will be evaluated using the selected measures detailed below, which have demonstrated reliability and validity.

#### Unmet needs

The Supportive Care Needs Survey - Partners and Caregivers (SCNS-P&C44) is a 44 item measure, which will be used to assess caregivers' level of unmet needs across the following domains: *Health Care Service Needs*, *Psychological and Emotional Needs*, *Work and Social Needs *and *Information Needs *[26].

#### Anxiety and depression

The Hospital Anxiety and Depression Scale (HADS), a 14-item survey with scoring that classifies participants' anxiety and depression levels as low, borderline or clinically significant [27], will be used to assess caregivers' level of anxiety and depression.

#### Quality of Life

QoL will be assessed using the SF-12v2, a 12-item measure evaluating QoL across 8 health concepts: physical functioning, role limitations because of physical health problems, bodily pain, general health perceptions, vitality (energy/fatigue), social functioning, role limitations because of emotional problems, and general mental health [28].

#### Coping

The Brief COPE will be used to assess coping strategies used by caregivers participating in the study. The Brief COPE is a 28-item survey consisting of 14 scales (2 items per scale): self-distraction, active coping, denial, substance use, use of emotional support, use of instrumental support, behavioural disengagement, venting, positive reframing, planning, humour, acceptance, religion, and self-blame [29].

### Sample size and statistical analysis

A sample of 520 caregivers will be recruited. This number has been estimated to account for approximately 25% attrition over the study period, so that 400 will complete the study, 200 in each group. This will involve approximately 330 GPs in total. The primary outcome measure is levels of unmet needs as measured by the SCNS-P&C. Based on previous research [24] utilising the standard deviations for each of the domains for the patient version of the SCNS [30-32] (between 15 to 24), and using a 5% significance level, having a minimum of 200 caregivers in each of the two comparison groups will give the study 80% power to detect a difference of between 4.2% and 6.7% in each of the SCNS-P&C domains for caregivers. It will also be able to detect a difference of between 0.9 and 1.4 units in anxiety and depression scores (HADS [7]), the secondary outcome measures.

Longitudinal analyses of the endpoints over time will be undertaken using generalised estimating equations (GEEs) [33]. GEEs are an extension to generalized linear models (GLMs). Linear regression models can only analyse the data cross-sectionally, however the 'extension' component of the GEE model allows for the analysis of the data longitudinally, thus reflecting the relationship between the longitudinal development of the dependent variable and the longitudinal development of the independent variables over time. An indicator variable identifying the control/intervention groups, socio-demographic factors and other confounding variables will be included in the GEE models. Analyses will be conducted on an intention to treat basis.

### Qualitative data collection and analysis

A sub-sample of up to 20 caregivers and 10 GPs will be interviewed following the study period (i.e. after the 6 month follow-up), using semi-structured interviews to identify caregivers' perceived ability to self-identify their needs and complete the NAT-C and GPs' perceptions of the usefulness of the GP Toolkit, to further inform broader implementation. The interview is expected to take approximately 20 minutes.

The qualitative interviews with GPs and caregivers will be transcribed and subjected to thematic analysis to obtain insights regarding the potential value of interventions initiated from general practice, and participants' perceptions of the usefulness of the resources. These results will enable the GP Toolkit resources to be refined before broader implementation.

## Discussion

There are several features of this study design, which take into account the unique challenges inherent in achieving adequate sample size in this difficult population and for a general practice intervention. The first is that recruitment has to be multi-site to achieve the required sample size, which requires collaboration between university-based investigators and groups of clinicians with different work practices. This requires differing recruitment methods being utilised at different sites. We believe this will not lead to recruitment bias because randomisation is a blinded process conducted off site from the recruiting hospitals. Any systematic differences in the patients recruited from the different sites will be distributed evenly between intervention and control groups.

Secondly, we have elected to break randomisation for subsequent patients of a participating GP. Allowing the one GP to treat both intervention and control patients would risk significant contamination and based on our previous experience [21,22], it is not feasible to recruit and randomise one patient per GP with the time and funding available to us through this grant. The randomisation procedure will ensure an even distribution of GPs with more than one patient in each group, minimising the impact of this variation in methods.

Thirdly, we are recruiting through oncology units, even though the intervention will take place in community general practices. This applies a very important principle in general practice-based research, which differs from specialty-based research. When the subjects of the research have a low prevalence condition, it is more feasible to recruit them from situations where the condition is prevalent, then recruit the GPs through the consenting participants. Asking GPs who constantly see a range of undifferentiated problems and many patients per day to recruit people with conditions they may see only a few times per annum is impractical. They are likely to forget that they are participating in the study and miss recruitment opportunities. This method was piloted by GM, and was successfully applied in a similar RCT of palliative care patients [21,22].

Finally, recruiting caregivers of patients with advanced cancer through oncology units means that patients who have progression to an incurable condition will be identified early. Caregivers will find the burden of care builds from that point. This recruitment method allows the intervention to have both a preventive effect and a treatment effect. Prevention can occur because potential problems are identified and anticipatory strategies initiated. Treatment occurs because existing problems are identified - many carers will otherwise not disclose them. The impact of this double effect may become more evident over a six-month period than an intervention applied in a palliative care service setting, where there will be less time for an intervention to have an impact.

This is the first intervention to specifically focus on addressing caregiver needs by using a consumer-oriented approach. This provides caregiver-specific data in a systematic way to their primary health care providers, in this case the caregiver's GP. This approach of providing feedback of patient-specific data to health care providers is feasible, acceptable to both patients and health care providers, and can alter the process and some outcomes of care [34]; indicating that the current study is not only logical but has the capacity to inform us as to what may or may not work in alleviating the unmet needs of caregivers in the general practice setting. Additionally, if the results of this study are favourable, we have the potential to influence the health care profession by providing an effective and achievable strategy that GPs can use to address the concerns of caregivers, therefore moving us one step closer in fulfilling the aims of a holistic approach to PC.

## Abbreviations

PC: Palliative Care; GP: General Practitioner; QoL: Quality of Life; CATI: Computer Assisted Telephone Interview; RCT: Randomized Control Trial; HADS: Hospital Anxiety and Depression Scale; NAT-C: Needs Assessment Tool - Caregivers; NAT: PD-C: Needs Assessment Tool: Progressive Disease - Cancer; SCNS-P&C: Supportive Care Needs Survey - Partners & Caregivers; GEE: Generalised Estimating Equation

## Competing interests

The authors declare that they have no competing interests.

## Authors' contributions

GM and AG developed the study concept and aims and initiated the project. MJ, DS and LB assisted in further development of the protocol. AG was responsible for drafting the manuscript. GM, LB and AG will implement the protocol and oversee collection of the data; and MJ undertook the pilot study. All authors contributed to the final manuscript.
